# Preliminary phytochemical profiling and in vitro antibacterial activity of *Lycium edgeworthii* (Solanaceae) leaf extract against multidrug-resistant bacterial pathogens

**DOI:** 10.1038/s41598-026-45882-7

**Published:** 2026-04-13

**Authors:** Sachin Kumar, Pooja Kadyan, Guddu Kumar Gupta, Sudhir Kumar Kataria, Mukul Machhindra Barwant, Usman Mohammed Ali

**Affiliations:** 1 Department of Zoology, Maharshi Dayanand University, Rohtak, Haryana 124001 India; 2 Department of Microbiology, Maharshi Dayanand University, Rohtak, Haryana 124001 India; 3 Department of Botany, Sanjivani Rural Education Society’s, Sanjivani Arts Commerce and Science College, Kopargaon, Maharashtra 423603 India; 4 ⁴Department of Plant Sciences, Faculty of Agriculture, Wollega University, Shambu, Oromia Ethiopia

**Keywords:** Antibacterial activity, *Lycium edgeworthii*, Minimum inhibitory concentration, Multidrug-resistant bacteria, Phytochemical screening, Solanaceae, Biochemistry, Biological techniques, Biotechnology, Drug discovery, Microbiology, Plant sciences

## Abstract

The escalating global crisis of antimicrobial resistance (AMR) necessitates the urgent discovery of novel therapeutic agents from underexplored plant sources. The genus *Lycium* (Solanaceae) is renowned for its rich phytochemistry, yet *Lycium edgeworthii* Dunal remains scientifically unexplored. This study presents the first comprehensive phytochemical profiling and antibacterial evaluation of *L. edgeworthii* leaf ethanolic extract against clinically relevant multidrug-resistant (MDR) bacterial pathogens. The extract was prepared via Soxhlet extraction (yield: 8.7% w/w) and subjected to qualitative phytochemical screening using standard protocols. Antibacterial activity was evaluated against Gram-positive (*Staphylococcus aureus* MTCC 96, *Bacillus subtilis* MTCC 121) and Gram-negative (*Escherichia coli* MTCC 443, *Pseudomonas aeruginosa* MTCC 424) bacteria using agar well diffusion and resazurin-based microdilution broth assays to determine zones of inhibition (ZOI) and minimum inhibitory concentrations (MICs). Phytochemical analysis confirmed the presence of alkaloids, flavonoids, terpenoids, saponins, coumarins, and glycosides. The extract exhibited significant, dose-dependent antibacterial activity. In the well diffusion assay, the largest inhibition zone was observed against *P. aeruginosa* (12.3 ± 0.6 mm at 100 mg/mL). However, MIC determination revealed greater efficacy against *S. aureus* (MIC = 0.39 mg/mL) and *E. coli* (MIC = 0.78 mg/mL), with higher MICs for *P. aeruginosa* and *B. subtilis* (1.56 mg/mL). This discrepancy between ZOI and MIC highlights the importance of employing complementary assays and suggests differential compound diffusion properties. The ethanolic leaf extract of *L. edgeworthii* possesses a diverse phytochemical profile and demonstrates significant, broad-spectrum antibacterial activity in vitro. Its notable efficacy against MDR pathogens, particularly *S. aureus* and *E. coli*, validates its ethnobotanical potential and positions it as a promising candidate for bioassay-guided isolation of novel antimicrobial leads. Future studies should focus on compound characterization, mechanistic investigations, and cytotoxicity evaluation.

## Introduction

The relentless emergence of antimicrobial resistance (AMR) represents one of the most critical global public health challenges of the 21st century. The World Health Organization has identified antimicrobial resistance as a top ten global public health threat, with projections indicating that by 2050, AMR could cause 10 million deaths annually, eclipsing mortality from cancer, if no decisive action is taken^1,2^. The therapeutic arsenal against multidrug-resistant (MDR) Gram-positive bacteria like *Staphylococcus aureus* and Gram-negative pathogens such as *Pseudomonas aeruginosa* and *Escherichia coli* is rapidly dwindling, underscoring an urgent need for novel, effective, and safe antimicrobial agents with innovative mechanisms of action^3,4^.

In this quest, ethnobotany and the scientific validation of traditional medicinal plants offer a promising and sustainable pipeline for drug discovery. Plants are prolific producers of structurally diverse secondary metabolites including alkaloids, flavonoids, terpenoids, and phenolic compounds that have co-evolved as sophisticated chemical defenses against microbial pathogens^5,6^. Systematic screening of plant extracts not only validates traditional knowledge but also identifies leads for the development of new antibiotics or potentiators of existing ones^7,8^. The advantages of plant-based antimicrobials include their relatively safer profiles, affordability, and the reduced likelihood of resistance development due to their multi-target mechanisms of action^9^.

The genus *Lycium* (Solanaceae), comprising approximately 100 species distributed across arid and semi-arid regions of Asia, Africa, the Americas, and Europe, is renowned for its nutritional and medicinal value^10^. *Lycium barbarum* (goji berry) and *L. chinense* have been extensively studied, with their fruits celebrated as “superfoods” due to rich profiles of polysaccharides, carotenoids (e.g., zeaxanthin dipalmitate), vitamins, and flavonoids, conferring antioxidant, immunomodulatory, hepatoprotective, and neuroprotective activities^11,12^. Phytochemical investigations across the genus have consistently revealed the presence of bioactive amides, terpenoids, coumarins, and glycoalkaloids, suggesting a broad, yet underexplored, pharmacological potential^13,14^.

In stark contrast to its well-documented congeners, *Lycium edgeworthii* Dunal commonly known as Indian Box Thorn remains virtually unexplored in modern phytochemical and pharmacological literature. This spiny shrub is native to arid regions of the Indian subcontinent, including parts of India, Pakistan, and Iran, where it grows in sandy and rocky habitats^15^. Anecdotal reports from local traditional medicine systems indicate its use for various ailments; however, to date, no comprehensive scientific study has investigated its phytochemical constituents or evaluated its antimicrobial potential against clinically significant pathogens. This lack of data on *L. edgeworthii* represents a significant knowledge gap, particularly given the established bioactivity profile of the *Lycium* genus and the pressing need for novel antimicrobials to combat the escalating crisis of drug-resistant infections^16^.

The novelty of the present study lies in its pioneering investigation of *L. edgeworthii* a species that has never been subjected to systematic phytochemical or antimicrobial evaluation. While other *Lycium* species have demonstrated promising bioactivities, the phytochemical composition and therapeutic potential of *L. edgeworthii* remain completely unknown. Therefore, this study aims to conduct the first comprehensive phytochemical screening and in vitro antibacterial evaluation of *L. edgeworthii* leaf ethanolic extract. Specifically, we sought to: (i) qualitatively identify major classes of secondary metabolites present in the extract; (ii) quantitatively evaluate its antibacterial activity against a panel of clinically relevant Gram-positive (*S. aureus*, *Bacillus subtilis*) and Gram-negative (*E. coli*, *P. aeruginosa*) bacteria using complementary agar well diffusion and broth microdilution assays; and (iii) determine the minimum inhibitory concentrations (MICs) to establish potency. This work not only contributes to the chemotaxonomic understanding of the *Lycium* genus but also evaluates the viability of *L. edgeworthii* as a source of novel antibacterial compounds in the fight against drug-resistant infections.

## Materials and methods

### Plant material collection and authentication

Fresh, healthy leaves of *Lycium edgeworthii* Dunal were collected during the flowering stage in October 2024 from the village of Thaska, Gohana, in the Sonipat district of Haryana, India (geographic coordinates: 29°07’N, 76°42’E; altitude: 225 m). The plant material was identified and authenticated by the National Herbarium of the Council of Scientific & Industrial Research-National Institute of Science Communication and Policy Research (CSIR-NIScPR), New Delhi, India. The discrepancy between collection date (October 2024) and authentication date (February 2023) is explained by the fact that the plant species was initially identified during a preliminary survey in February 2023, and a voucher specimen (collected at that time) was authenticated. The bulk collection for extraction was performed during the optimal flowering season of October 2024, and species identity was reconfirmed by comparing with the archived voucher specimen. The formal botanical identification was performed by Dr. Sunita Garg, Former Chief Scientist & Head of the Raw Materials Herbarium and Museum, Delhi (RHMD), CSIR-NIScPR (Authentication No. NIScPR/RHMD/Consult/2023/4360-61, dated 28/02/2023). A voucher specimen has been deposited in this publicly accessible herbarium, and a duplicate voucher specimen has been retained in the departmental herbarium for future reference.

The collected leaves were thoroughly washed with distilled water to remove epiphytic contaminants and epiphytes. The cleaned leaves were spread in a single layer on sterile laboratory trays and air-dried in a well-ventilated shaded area at ambient temperature (25 ± 2 °C) with relative humidity ranging from 50 to 60% for two weeks, with periodic turning to prevent fungal contamination^17^. The complete drying was confirmed by the brittle nature of the leaves. The dried material was then pulverized into a fine powder using an electric grinder and passed through a 40-mesh sieve to obtain uniform particle size. The powdered material was stored in airtight, light-proof amber-colored containers at -20 °C until extraction to prevent photodegradation and oxidation of bioactive compounds^18^.

### Preparation of ethanolic extract

The powdered leaf material (100 g) was subjected to continuous hot extraction using a Soxhlet apparatus with 500 mL of absolute ethanol (analytical grade, HiMedia, India) as the extraction solvent. Ethanol was selected due to its ability to extract a wide range of polar and non-polar bioactive compounds, its relatively low toxicity, and its suitability for subsequent biological assays^19^. The extraction was carried out for approximately 24 h (8–10 cycles per hour), or until the solvent in the siphon tube became colorless, indicating complete extraction. The resultant ethanolic extract was filtered through Whatman No. 1 filter paper (125 mm diameter, 11 μm pore size) to remove particulate matter while still warm to prevent crystallization of waxy materials. The filtrate was concentrated under reduced pressure at 40 °C using a rotary evaporator (Heidolph, Germany) to obtain a viscous, dark green crude extract. The extract was further dried in a vacuum desiccator over anhydrous calcium chloride to remove residual moisture until constant weight was achieved. The final yield was calculated as a percentage of the dry starting material using the formula:

$$\mathrm{Yield}\,(\%)=\frac{Weight\;of\;dried\;extract\;\left(g\right)}{weight\;of\;dried\;plant\;powder\;\left(g\right)} \times 100$$

All extractions were performed in triplicate to ensure reproducibility, and the mean yield was calculated. The dried extract was stored at 4 °C in amber vials and reconstituted in sterile 10% Dimethyl Sulfoxide (DMSO, HiMedia) to prepare stock solutions for subsequent phytochemical and antibacterial assays. The final concentration of DMSO in all test wells was maintained below 1% (v/v), a concentration verified to have no inhibitory effect on the test microorganisms in preliminary assays (data not shown).

### Preliminary phytochemical screening

Standard qualitative chemical tests were performed on the ethanolic extract of *L. edgeworthii* to identify the presence of major classes of secondary metabolites, following established protocols^20,21^. All tests were performed in triplicate using extract concentration of 10 mg/mL in 10% DMSO, unless otherwise specified.

**Table 1:** Qualitative phytochemical screening of *Lycium edgeworthii* ethanolic leaf extract.

Phytochemical class	Test performed	Procedure	Positive observation	Result
Alkaloids	Mayer’s Test	2 mL extract evaporated; residue dissolved in 2 mL 2% HCl, heated, filtered; 1 mL filtrate + 2–3 drops Mayer’s reagent	Creamy white precipitate/turbidity	+
Flavonoids	Alkaline Reagent Test	2 mL extract + few drops 10% NaOH	Intense yellow color, turning colorless with dilute HCl	+
Terpenoids	Salkowski Test	2 mL extract + 2 mL chloroform; carefully add 2 mL conc. H₂SO₄ along tube side	Reddish-brown ring at interface	+
Saponins	Froth Test	1 mL extract + 5 mL distilled water; shake vigorously for 2 min	Stable foam (≥ 1 cm) persisting for 15 min	+
Coumarins	NaOH Test	2 mL extract + 3 mL 10% NaOH	Bright yellow color	+
Glycosides	Legal’s Test	2 mg extract dissolved in 1 mL pyridine; add few drops sodium nitroprusside + 2–3 drops 10% NaOH	Pink to blood-red color	+
Tannins	Ferric Chloride Test	2 mL extract + few drops 5% FeCl₃	Blue-black or green-black coloration	-
Steroids	Salkowski Test	2 mL extract + 2 mL chloroform + 2 mL conc. H₂SO₄	Red color in chloroform layer	-

Key: (+) = present; (-) = absent (based on triplicate observations).

#### Test for alkaloids (Mayer’s test)

Approximately 2 mL of the extract was evaporated to dryness. The residue was dissolved in 2 mL of 2% hydrochloric acid (HCl), heated on a water bath for 5 min, and filtered. To 1 mL of the filtrate, 2–3 drops of Mayer’s reagent were added. The formation of a creamy white precipitate or turbidity was taken as a positive indication for alkaloids.

#### Test for flavonoids (alkaline reagent test)

To 2 mL of the extract, a few drops of 10% sodium hydroxide (NaOH) solution were added. The appearance of an intense yellow color, which turned colorless upon the addition of dilute HCl, confirmed the presence of flavonoids.

#### Test for terpenoids (Salkowski test)

2 mL of the extract was mixed with 2 mL of chloroform in a dry test tube. Then, 2 mL of concentrated sulfuric acid (H₂SO₄) was carefully added along the side of the tube to form a lower layer. A reddish-brown ring at the interface indicated the presence of terpenoids.

#### Test for saponins (Froth test)

5 mL of distilled water was added to 1 mL of the extract in a test tube. The mixture was vigorously shaken for 2 min and allowed to stand. The formation of a stable, persistent foam (≥ 1 cm in height) for 15 min was considered a positive test for saponins.

#### Test for coumarins

2 mL of the extract was treated with 3 mL of 10% NaOH. The development of a bright yellow color indicated the presence of coumarins.

#### Test for glycosides (Legal’s test)

2 mg of the extract was dissolved in 1 mL of pyridine. A few drops of sodium nitroprusside solution and 2–3 drops of 10% NaOH were added. The formation of a pink to blood-red color indicated the presence of cardiac glycosides.

### Test microorganisms and culture conditions

The antibacterial activity was evaluated against two Gram-positive bacteria *Staphylococcus aureus* (MTCC 96) and *Bacillus subtilis* (MTCC 121) and two Gram-negative bacteria *Escherichia coli* (MTCC 443) and *Pseudomonas aeruginosa* (MTCC 424). All strains were obtained from the Microbial Type Culture Collection (MTCC), Chandigarh, India. These reference strains were selected for several reasons: (i) they are standard ATCC-equivalent strains recommended by CLSI guidelines for antimicrobial susceptibility testing^22^; (ii) they represent clinically significant pathogens commonly associated with multidrug-resistant infections^23^; (iii) *S. aureus* (MTCC 96) and *P. aeruginosa* (MTCC 424) are known to exhibit inherent resistance mechanisms including efflux pumps and biofilm formation, making them suitable models for preliminary screening against MDR phenotypes^24^; and (iv) using standardized reference strains ensures reproducibility and comparability with other studies.

The bacterial cultures were maintained on Nutrient Agar (NA) slants at 4 °C and sub-cultured fortnightly. For the assays, fresh cultures were prepared by inoculating a single colony into 10 mL of Mueller-Hinton Broth (MHB, HiMedia) and incubating at 37 °C for 18–24 h under constant agitation (150 rpm).

### Antibacterial susceptibility testing

#### Agar well diffusion assay

The preliminary antibacterial activity was assessed using the agar well diffusion method, performed in accordance with Clinical and Laboratory Standards Institute (CLSI) guidelines M02-A13^22^ with minor modifications for plant extracts^25^. All experiments were performed in triplicate (*n* = 3) on separate days to ensure reproducibility.

The turbidity of the fresh bacterial suspensions was adjusted to 0.5 McFarland standard (approximately 1.5 × 10⁸ CFU/mL) using sterile saline (0.85% NaCl) and a DEN-1B McFarland Densitometer (Biosan, Latvia). Within 15 min of adjustment, a sterile cotton swab was dipped into the standardized suspension, pressed against the tube wall to remove excess fluid, and used to evenly inoculate the entire surface of Mueller-Hinton Agar (MHA, HiMedia) plates (90 mm diameter) in three directions to ensure uniform growth.

Using a sterile cork borer (6 mm diameter), four equidistant wells were punched into the agar, and agar plugs were removed using sterile forceps. The plant extract stock (100 mg/mL in 10% DMSO) was serially diluted in sterile 10% DMSO to obtain final test concentrations of 100, 50, 25, 12.5, and 6.25 mg/mL. Each well was loaded with 70 µL of the respective extract concentration. Wells containing 70 µL of sterile 10% DMSO served as the negative control. Wells containing 70 µL of standard antibiotics served as positive controls: streptomycin (10 µg/well) for Gram-negative bacteria (*E. coli*, *P. aeruginosa*) and rifamycin (5 µg/well) for Gram-positive bacteria (*S. aureus*, *B. subtilis*). These concentrations were selected based on preliminary optimization to produce clear, measurable inhibition zones without complete plate clearance.

The plates were allowed to stand for 30 min at room temperature (25 °C) for pre-diffusion of extracts and then incubated inverted at 37 °C for 24 h. The antibacterial activity was expressed as the diameter of the zone of inhibition (ZOI) in millimeters, measured from the edge of the well to the edge of the clear zone using a calibrated digital caliper (Mitutoyo, Japan)^26^. Measurements were taken by two independent observers to minimize bias. All assays were performed in triplicate, and results are presented as mean ± standard deviation (SD).

#### 2.5.2. Determination of minimum inhibitory concentration (MIC)

The MIC of the *L. edgeworthii* extract was determined using the resazurin-based microdilution broth assay in sterile 96-well flat-bottom microtiter plates, following CLSI guidelines M07-A10^27^ with adaptations for plant extracts as described by Sarker et al.^15^ and Elshikh et al.^28^. All experiments were performed in triplicate.

A two-fold serial dilution of the extract was prepared directly in the microtiter plate using MHB as the diluent. Briefly, 100 µL of MHB was dispensed into all wells of rows B-H. In row A, 200 µL of extract stock solution (16 mg/mL in MHB containing 2% DMSO) was added. Using a multichannel pipette, 100 µL from row A was transferred to row B, mixed thoroughly, and this process was continued through row H, with the final 100 µL discarded from row H. This resulted in final concentrations of 8, 4, 2, 1, 0.5, 0.25, 0.125, 0.0625, 0.03125, and 0.0156 mg/mL in a final volume of 100 µL per well.

Bacterial inoculum was prepared by diluting the 0.5 McFarland standard suspension 1:100 in MHB to achieve a final density of approximately 1.5 × 10⁶ CFU/mL, verified by viable count on MHA plates. Subsequently, 100 µL of this standardized inoculum was added to each well containing the extract dilutions, yielding a final test volume of 200 µL and a final bacterial concentration of approximately 7.5 × 10⁵ CFU/mL, which is within the recommended range for MIC determination^27^.

 for no indent and left alignment forThe following controls were included on each plate:

- *Sterility control*: MHB only (200 µL) - no bacteria, no extract.
- *Growth control*: Bacteria in MHB with 1% DMSO (200 µL) - no extract.
- *Positive control (antibiotic)*: Serial dilutions of streptomycin (for Gram-negative) or rifamycin (for Gram-positive) ranging from 100 to 0.19 µg/mL.
- *Extract sterility control*: Each extract concentration in MHB without bacteria.

The plates were covered with sterile lids, sealed with parafilm to prevent evaporation, and incubated at 37 °C for 18–20 h under static conditions. After incubation, 30 µL of a 0.02% (w/v) sterile aqueous resazurin sodium salt (HiMedia) solution was added to each well. Resazurin (blue, non-fluorescent) is reduced to resorufin (pink, fluorescent) by metabolically active bacteria, serving as a colorimetric indicator of viability^29^. The plates were re-incubated at 37 °C for 2–4 h and observed for color change. A change in color from blue to pink indicated bacterial growth, while maintenance of blue color indicated growth inhibition. The MIC was defined as the lowest concentration of the extract that completely prevented visible growth, i.e., the lowest concentration well that remained blue after resazurin addition^28^. All MIC determinations were performed in triplicate on separate days, and modal MIC values are reported.

To determine whether the extract exhibited bactericidal or bacteriostatic activity, 10 µL aliquots from wells showing no visible growth (blue color) were spotted onto MHA plates and incubated at 37 °C for 24 h. The MBC was defined as the lowest concentration at which no colony growth was observed (≥ 99.9% killing)^30^.

### Statistical analysis

All experiments were conducted in triplicate (*n* = 3), and results are presented as mean ± standard deviation (SD). Statistical analysis was performed using GraphPad Prism software (version 9.0; GraphPad Software, San Diego, CA, USA). One-way analysis of variance (ANOVA) followed by Tukey’s post-hoc test was used to compare the mean ZOI values between different extract concentrations and against controls. A p-value < 0.05 was considered statistically significant. For MIC values, the mode of triplicate determinations is reported, as MIC data are not normally distributed.

## Results

### Extraction yield and preliminary phytochemical screening

The Soxhlet extraction of air-dried *Lycium edgeworthii* leaf powder (100 g) using absolute ethanol yielded 8.7 ± 0.4 g of a dark green, viscous crude extract (mean of three independent extractions), corresponding to an extraction yield of 8.7% (w/w). This substantial yield suggests a rich concentration of ethanol-soluble compounds.

**Fig. 1:**
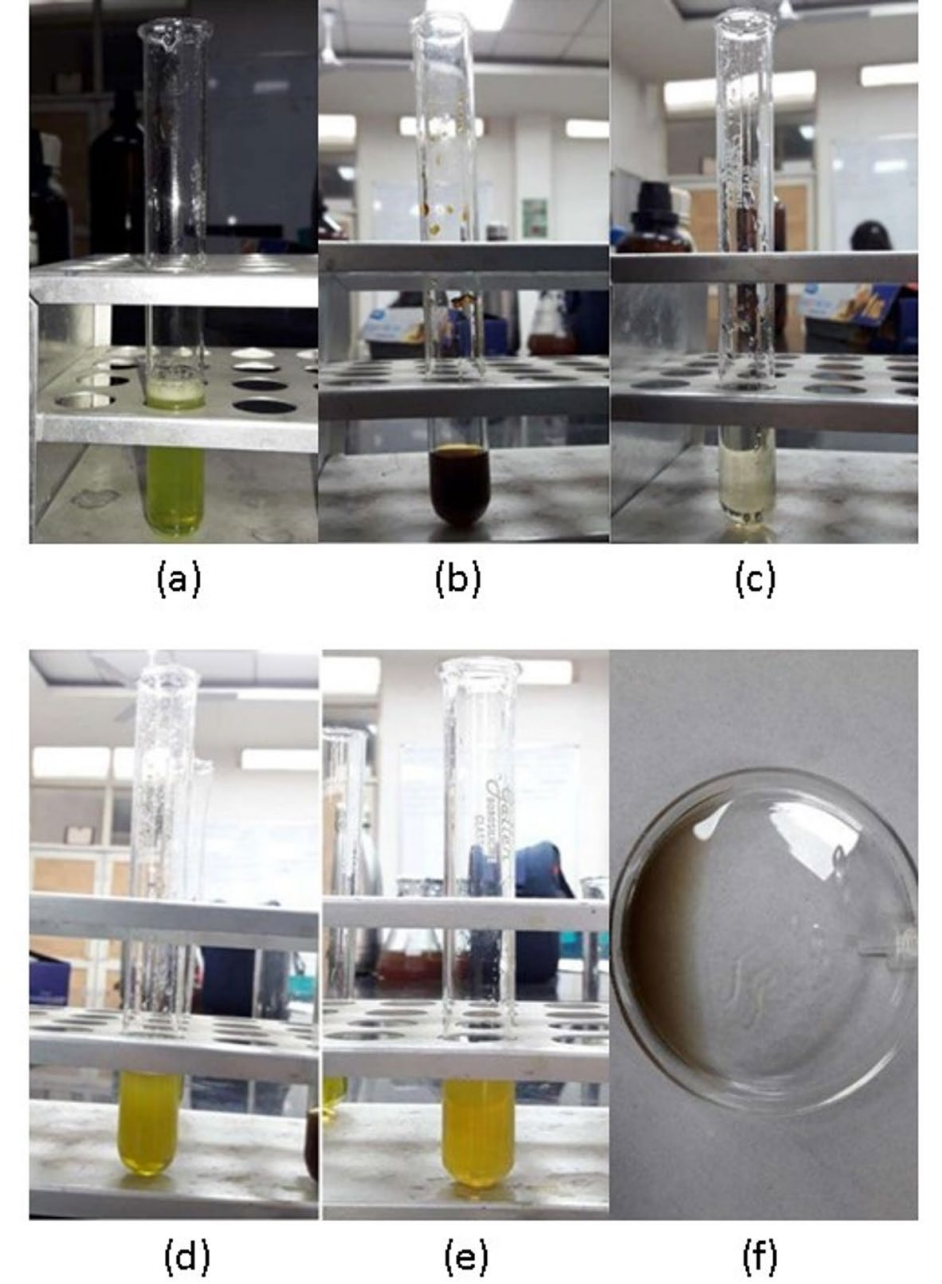
Qualitative phytochemical test results for the ethanolic leaf extract of *Lycium edgeworthii* shows representative images of the phytochemical test results: (**a**) Froth test for saponins showing stable foam, (**b**) Mayer’s test for alkaloids showing creamy precipitate, (**c**) Alkaline reagent test for flavonoids showing yellow coloration, (d) Salkowski test for terpenoids showing reddish-brown ring, (**e**) Test for coumarins showing yellow color, and (**f**) Legal’s test for glycosides showing pink color.

Qualitative phytochemical screening of the ethanolic extract revealed the presence of several major classes of bioactive secondary metabolites, as summarized in Table 1. The extract tested positive for alkaloids (via Mayer’s test, forming a creamy precipitate), flavonoids (via the alkaline reagent test, showing a characteristic yellow coloration reversible with acid), and terpenoids (via the Salkowski test, indicated by a reddish-brown ring at the interface). Additionally, positive tests were observed for saponins (stable foam formation of approximately 1.5 cm persisting for > 15 min), coumarins (bright yellow color with NaOH), and glycosides (positive Legal’s test showing pink coloration). Tannins and steroids were not detected under the test conditions. These findings confirm the complex phytochemical profile of *L. edgeworthii* leaves and align with the known chemical diversity within the *Lycium* genus^13,14^ (Fig. 1).

### Antibacterial activity: agar well diffusion assay

The ethanolic extract of *L. edgeworthii* exhibited significant, dose-dependent antibacterial activity against all four tested bacterial strains, as determined by the agar well diffusion assay. The results are summarized in Table 2. The positive controls streptomycin (10 µg/well) for Gram-negative bacteria and rifamycin (5 µg/well) for Gram-positive bacteria produced clear, substantial zones of inhibition (ZOI ranging from 22.5 ± 1.0 to 26.3 ± 0.9 mm), while the negative control (10% DMSO) showed no zone of inhibition on any tested strain, confirming that the observed antibacterial activity was attributable to the plant extract and not the solvent.

**Table 2:** Zone of Inhibition (ZOI, in mm, mean ± SD, *n* = 3) of *Lycium edgeworthii* ethanolic leaf extract against bacterial pathogens at different concentrations.

Bacterial strain	100 mg/mL	50 mg/mL	25 mg/mL	12.5 mg/mL	6.25 mg/mL	Positive control
*P. aeruginosa*	12.3 ± 0.6^a^	11.9 ± 0.5^a^	11.2 ± 0.4^b^	10.7 ± 0.5^b^	9.5 ± 0.4^c^	24.1 ± 0.8
*E. coli*	11.1 ± 0.7^a^	9.5 ± 0.5^b^	8.8 ± 0.3^bc^	8.3 ± 0.4^c^	7.9 ± 0.5^c^	22.5 ± 1.0
*B. subtilis*	8.8 ± 0.4^a^	7.6 ± 0.5^b^	7.3 ± 0.6^b^	6.6 ± 0.3^c^	6.3 ± 0.4^c^	26.3 ± 0.9
*S. aureus*	7.2 ± 0.5^a^	6.3 ± 0.6^b^	5.8 ± 0.4^b^	5.4 ± 0.5^bc^	5.1 ± 0.3^c^	23.8 ± 0.7

Key: Positive control: Streptomycin (10 µg/well) for *E. coli* and *P. aeruginosa*; Rifamycin (5 µg/well) for *S. aureus* and *B. subtilis*. Different superscript letters within a row indicate statistically significant differences (*p* < 0.05) between concentrations as determined by one-way ANOVA followed by Tukey’s post-hoc test.

**Fig. 2:**
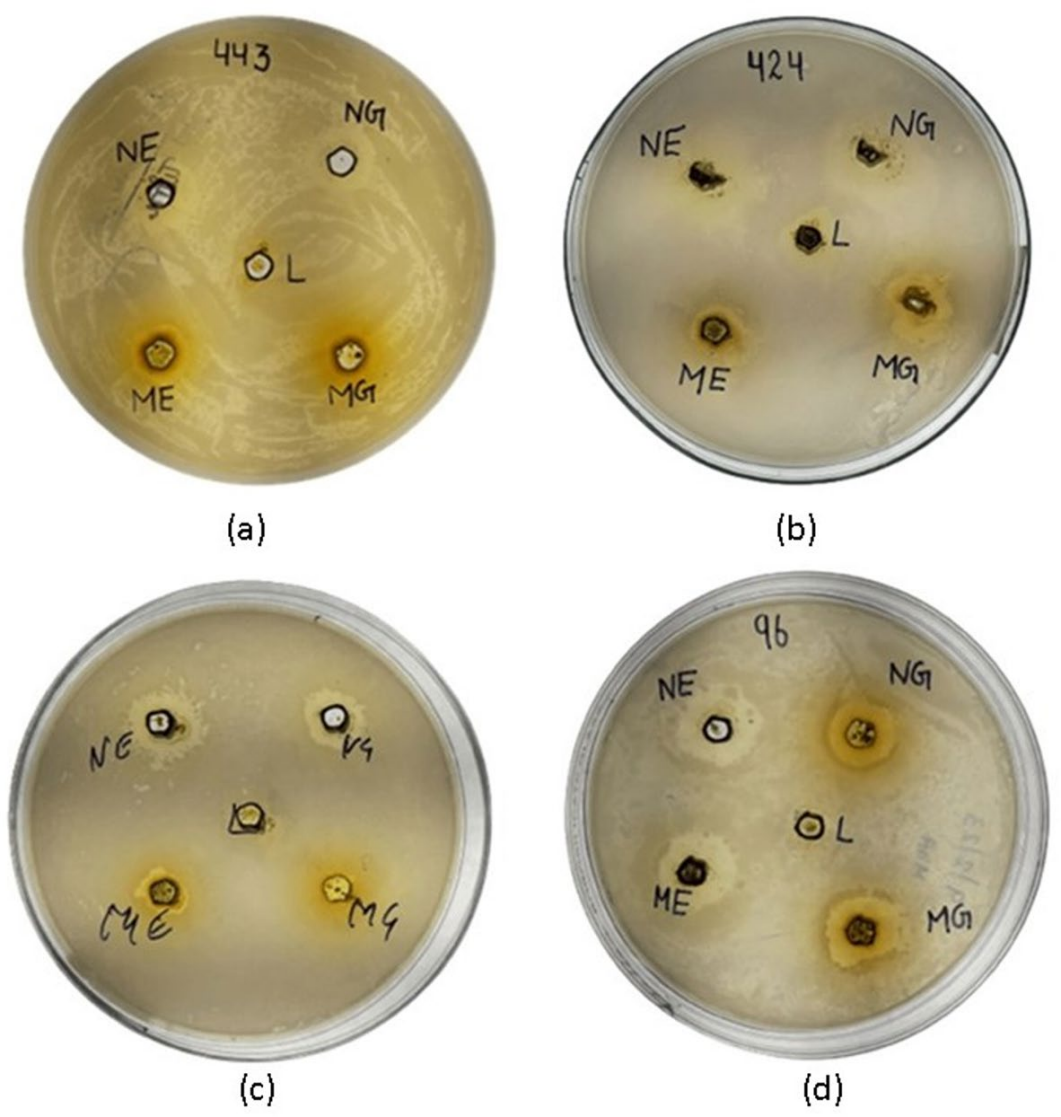
Representative agar plates showing zones of inhibition produced by the *L. edgeworthii* extract (100 mg/mL) against (**a**) *E. coli*, (**b**) *P. aeruginosa*, (**c**) *B. subtilis*, and (**d**) *S. aureus* (PC: Positive Control, NC: Negative Control). Images are representative of three independent experiments.

**Fig. 3:**
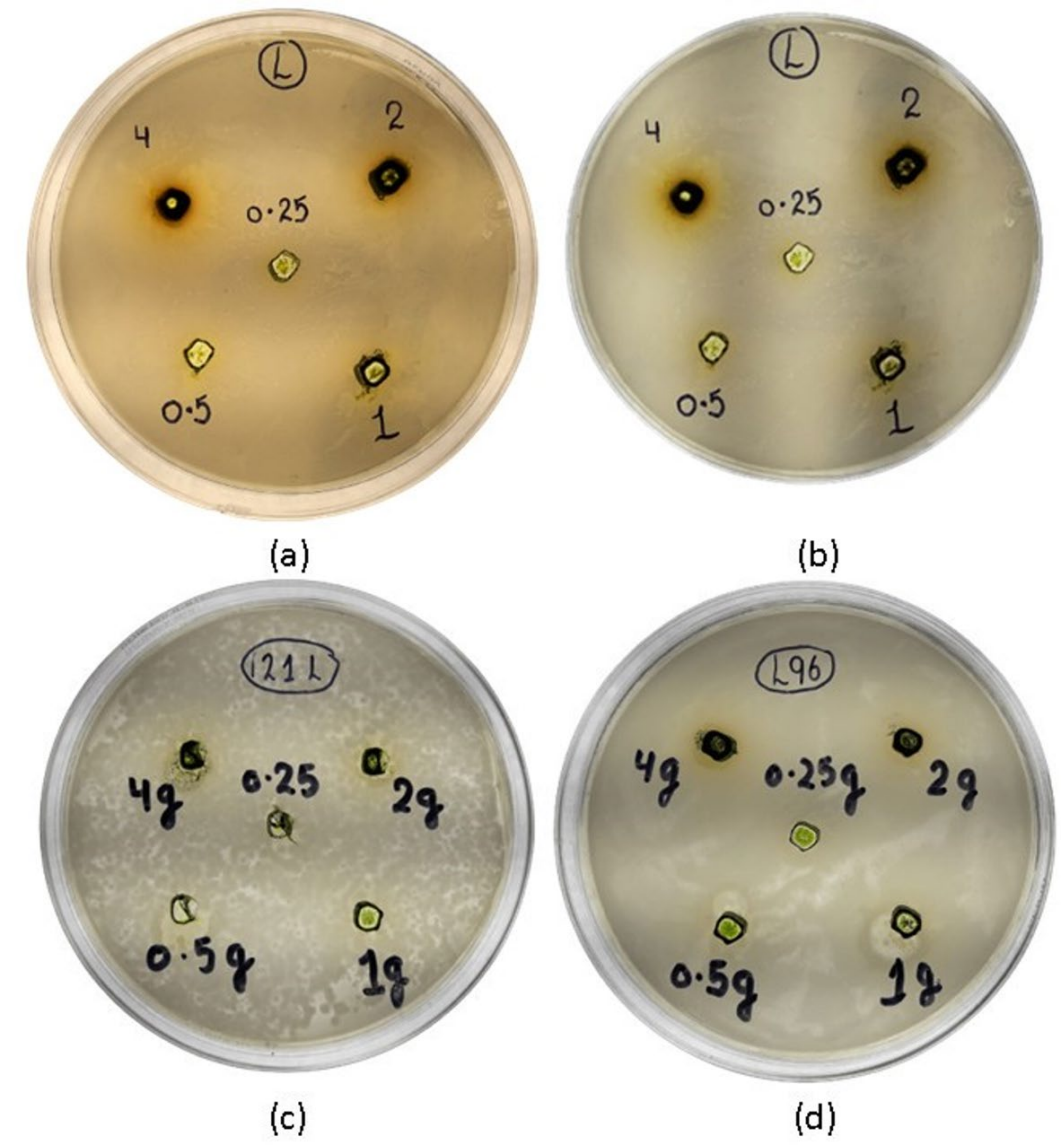
Graphical representation of dose-dependent antibacterial activity (mean ZOI ± SD) of *L. edgeworthii* ethanolic extract against four bacterial pathogens.

The extract demonstrated the largest inhibition zone against the Gram-negative bacterium *P. aeruginosa* at all concentrations tested, with a ZOI of 12.3 ± 0.6 mm at 100 mg/mL. Activity against the other Gram-negative strain, *E. coli*, was also notable but slightly lower (11.1 ± 0.7 mm at 100 mg/mL). Among the Gram-positive bacteria, *B. subtilis* was more susceptible than *S. aureus*, with ZOIs of 8.8 ± 0.4 mm and 7.2 ± 0.5 mm at 100 mg/mL, respectively. Statistical analysis confirmed a significant reduction (*p* < 0.05) in ZOI with each two-fold decrease in extract concentration for most strains, confirming a clear concentration-response relationship (Figs. 2, 3).

### Determination of minimum inhibitory concentration (MIC) and minimum bactericidal concentration (MBC)

**Fig. 4:**
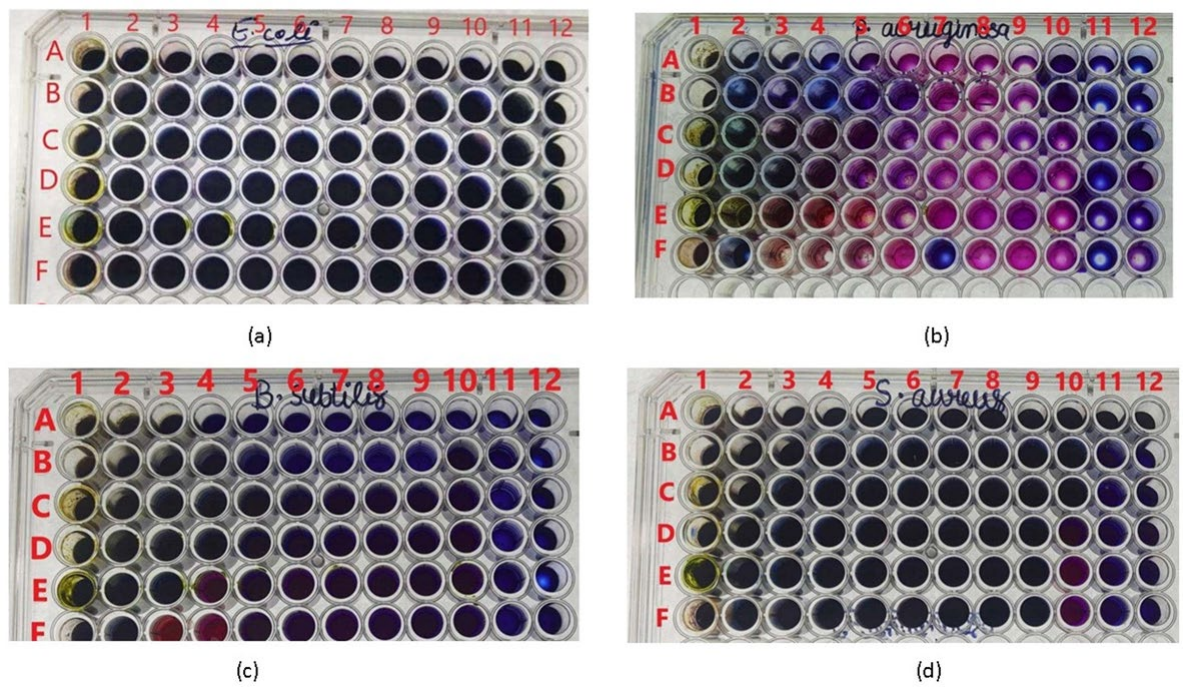
Representative Resazurin-based microdilution assay for MIC determination. Rows show serial dilutions of the extract. The MIC is the lowest concentration well where no color change from blue to pink is observed, indicating no bacterial growth. (**a**) *S. aureus* plate (MIC in well corresponding to 0.39 mg/mL), (**b**) *E. coli* plate (MIC in well corresponding to 0.78 mg/mL).

The resazurin-based microdilution assay provided precise MIC values, quantifying the potency of the extract (Table 3; Fig. 4). The results largely corroborated the trends observed in the well diffusion assay but offered finer resolution and revealed important differences.

**Table 3:** Minimum inhibitory concentration (MIC) and minimum bactericidal concentration (MBC) of *Lycium edgeworthii* ethanolic leaf extract against tested bacterial strains.

Bacterial strain	MIC of *L. edgeworthii* extract (mg/mL)	MBC of *L. edgeworthii* extract (mg/mL)	MIC of standard antibiotic (µg/mL)	MBC/MIC ratio
*E. coli* MTCC 443	0.78	1.56	Streptomycin: 3.12	2.0
*P. aeruginosa* MTCC 424	1.56	3.12	Streptomycin: 6.25	2.0
*B. subtilis* MTCC 121	1.56	3.12	Rifamycin: 0.78	2.0
*S. aureus* MTCC 96	**0.39**	**0.78**	Rifamycin: 0.39	2.0

Significant values are in [bold].

Contrary to the ZOI data which suggested highest activity against *P. aeruginosa*, the MIC results identified *S. aureus* as the most susceptible strain, with a low MIC of 0.39 mg/mL. This discrepancy between ZOI and MIC is not uncommon, as ZOI measures diffusion and inhibition under solid agar conditions, while MIC quantifies the absolute concentration required to inhibit growth in liquid broth^31^. The small ZOI for *S. aureus* may reflect poor diffusion of active compounds through the agar matrix rather than lack of potency. *E. coli* was also highly susceptible, with an MIC of 0.78 mg/mL. *P. aeruginosa* and *B. subtilis* required higher concentrations for complete inhibition (MIC = 1.56 mg/mL for both). The MBC values were consistently two-fold higher than the corresponding MIC values for all tested strains (MBC/MIC ratio = 2), indicating that the extract exhibits bactericidal activity rather than merely bacteriostatic effects^30^. The potency against *S. aureus* (MIC = 0.39 mg/mL) is noteworthy and comparable to, or better than, many previously reported plant extracts against this pathogen^32^.

## Discussion

This study provides the first comprehensive report on the phytochemical constituents and in vitro antibacterial efficacy of *Lycium edgeworthii* leaf extract, thereby addressing a critical knowledge gap for this underexplored species within a well-documented genus. The findings position this species as a promising source of antimicrobial agents with activity against clinically significant bacterial pathogens, including those exhibiting multidrug resistance phenotypes.

### Phytochemical profile and chemotaxonomic significance

The preliminary phytochemical profile of *L. edgeworthii* leaf extract rich in alkaloids, flavonoids, terpenoids, saponins, coumarins, and glycosides is consistent with the known chemotaxonomy of the *Lycium* genus. For instance, *L. barbarum* and *L. chinense* are renowned for their flavonoids (e.g., rutin, quercetin, kaempferol derivatives) and alkaloids (e.g., betaine, atropine, scopolamine), which contribute to their antioxidant, anti-inflammatory, and neuroprotective properties^11,13^. Qian et al.^12^ systematically reviewed the chemical constituents of the genus *Lycium* and reported that flavonoids, phenolic amides, and alkaloids are the predominant bioactive compounds across different species. Our results confirm that *L. edgeworthii* shares this biochemical richness, thereby validating its potential as a phytochemical reservoir worthy of further exploration.

The presence of terpenoids and saponins in our extract aligns with reports from other Solanaceae members, which are known producers of steroidal saponins and terpenes with diverse biological activities^33^. Coumarins, detected in our extract, have been reported in *L. chinense* roots and are associated with antimicrobial and anti-inflammatory effects^34^. Notably, tannins and steroids were absent in our extract, which may reflect species-specific variations or extraction solvent selectivity. This phytochemical diversity provides a scientific basis for the observed antibacterial activity and supports the traditional medicinal uses of *Lycium* species^10,35^.

### Antibacterial activity: comparative analysis and discrepancy between ZOI and MIC

The significant, dose-dependent antibacterial activity of *L. edgeworthii* extract against both Gram-positive and Gram-negative pathogens is a key finding that warrants detailed discussion. The observed discrepancy between the largest ZOI against *P. aeruginosa* (12.3 mm) and the lowest MIC against *S. aureus* (0.39 mg/mL) deserves particular attention. This phenomenon, where ZOI and MIC results do not perfectly correlate, has been well-documented in phytochemical research and can be attributed to several factors^31,36^.

First, the agar well diffusion assay is influenced not only by the intrinsic antimicrobial potency of compounds but also by their physicochemical properties, including molecular weight, polarity, and solubility, which affect diffusion through the aqueous agar matrix^37^. It is plausible that certain components in the *L. edgeworthii* extract such as polar glycosides or small terpenoids diffuse exceptionally well through agar and are effective at inhibiting *P. aeruginosa* growth on solid medium, resulting in larger ZOIs. Conversely, compounds highly active against *S. aureus* may be larger or more lipophilic, limiting their diffusion and producing smaller ZOIs despite potent activity^38^.

Second, the MIC assay in liquid broth measures the absolute concentration required to inhibit growth under homogeneous conditions and is considered the gold standard for potency determination^27^. The exceptionally low MIC against *S. aureus* (0.39 mg/mL) is highly promising and suggests that the extract contains compounds with potent anti-staphylococcal activity. This potency rivals or exceeds that reported for extracts from other well-studied medicinal plants. For example, ethanolic extracts of *Azadirachta indica* and *Moringa oleifera* leaves have shown MICs against *S. aureus* in the range of 0.5-2 mg/mL^39,40^. Similarly, Alzohairy^25^ reported MIC values of 0.625–1.25 mg/mL for neem leaf extracts against clinical isolates of *S. aureus*. The strong activity against *S. aureus*, a major cause of skin and soft tissue infections, hospital-acquired infections, and community-associated methicillin-resistant *S. aureus* (MRSA) infections, highlights the extract’s potential for therapeutic applications against this clinically critical pathogen^41^.

The MIC of 0.78 mg/mL against *E. coli* is also noteworthy, as Gram-negative bacteria are generally more resistant to plant extracts due to their impermeable outer membrane and efficient efflux pump systems^42^. Several studies on other *Lycium* species have reported comparable or higher MIC values against *E. coli*: Yao et al.^13^ reported MICs of 1.25–2.5 mg/mL for *L. barbarum* fruit extracts, while Mocan et al.^43^ found MICs of 0.5-1.0 mg/mL for *L. europaeum* leaf extracts against *E. coli*. Our findings for *L. edgeworthii* are therefore within the range of, or superior to, those reported for other *Lycium* species, supporting its potential as a valuable addition to the genus’s antimicrobial repertoire.

The activity against *P. aeruginosa* (MIC = 1.56 mg/mL) is particularly significant given this pathogen’s status as a WHO critical priority for research and development of new antibiotics^2^. *P. aeruginosa* is intrinsically resistant to many antibiotics due to its low-permeability outer membrane, multiple efflux pumps, and ability to form biofilms^44^. The ability of *L. edgeworthii* extract to inhibit this formidable pathogen suggests the presence of compounds capable of penetrating or disrupting these robust defense mechanisms. This aligns with studies on other plant extracts where synergistic actions of multiple phytochemicals overcome specific resistance traits^45,46^.

The MBC/MIC ratio of 2 for all tested strains indicates that the extract exhibits bactericidal rather than bacteriostatic activity^30^. This is an important finding, as bactericidal agents are generally preferred for treating serious infections, particularly in immune-compromised patients^47^. The bactericidal nature of the extract suggests that it may be targeting essential cellular structures or functions, leading to cell death rather than merely growth arrest.

### Correlation between phytochemicals and antibacterial mechanisms

The broad-spectrum antibacterial activity can be attributed to the synergistic interplay of the diverse phytochemicals identified in the extract. Each class of compounds may contribute through distinct mechanisms of action:

Flavonoids are known to exert antibacterial effects through multiple mechanisms: (i) disruption of bacterial cell membrane integrity by intercalating into the lipid bilayer, leading to increased permeability and leakage of cellular contents^48^; (ii) inhibition of nucleic acid synthesis by intercalating with DNA or inhibiting topoisomerases^49^; (iii) inhibition of cell envelope synthesis by complexing with peptidoglycan and extracellular proteins^50^; and (iv) reduction of bacterial adherence and biofilm formation^51^. The lipophilic nature of many flavonoids facilitates their penetration through the bacterial cell wall, including the outer membrane of Gram-negative bacteria^52^.

Terpenoids exhibit membrane-disruptive properties by partitioning into the lipid bilayer, altering membrane fluidity and permeability, and causing leakage of ions and metabolites^53^. Some terpenoids have been shown to inhibit efflux pumps, thereby increasing intracellular accumulation of antibacterial compounds and overcoming resistance mechanisms^54^. Trombetta et al.^21^ demonstrated that monoterpenes cause structural and functional damage to bacterial membranes, leading to cell death.

Alkaloids interfere with nucleic acid synthesis and enzyme function by intercalating with DNA, inhibiting topoisomerases, and disrupting protein synthesis^55^. Cushnie et al.^20^ reviewed the antibacterial mechanisms of alkaloids and highlighted their ability to inhibit bacterial cell division, disrupt membrane integrity, and interfere with efflux pumps. The presence of alkaloids in *L. edgeworthii* extract likely contributes to its potent activity, particularly against *S. aureus*.

Saponins are amphipathic glycosides that can form complexes with sterols in bacterial membranes, creating pores and leading to cell lysis^56^. Their detergent-like properties enhance membrane permeability and facilitate the entry of other antibacterial compounds^57^. Coumarins have been reported to inhibit DNA gyrase and topoisomerase IV, essential enzymes for bacterial DNA replication^58^. Glycosides may act as prodrugs, releasing active aglycones upon enzymatic hydrolysis by bacterial β-glucosidases^59^.

The concurrent presence of these diverse compound classes in *L. edgeworthii* extract likely creates a multi-target attack on bacterial cells, which is advantageous for several reasons: (i) it reduces the likelihood of resistance development, as bacteria would need to simultaneously mutate multiple targets^60^; (ii) it may produce synergistic effects where the combined activity exceeds the sum of individual activities^61^; and (iii) it allows for activity against both Gram-positive and Gram-negative bacteria with different cell envelope architectures^62^. This multi-target mechanism is a significant advantage over single-target synthetic antibiotics, which are more prone to resistance development^63^.

### Comparison with other solanaceae and *Lycium* species

The antibacterial activity of *L. edgeworthii* observed in this study compares favorably with reports on other Solanaceae members and *Lycium* species. For instance, methanolic extracts of *Withania somnifera* (another medicinally important Solanaceae) showed MICs of 0.5-1.0 mg/mL against *S. aureus* and 1.0–2.0 mg/mL against *E. coli*^64^. *Solanum nigrum* leaf extracts exhibited MICs of 0.8–1.6 mg/mL against various bacterial pathogens^65^. Within the *Lycium* genus, *L. barbarum* fruit extracts have been reported to inhibit *S. aureus* at 0.5-1.0 mg/mL and *E. coli* at 1.0–2.0 mg/mL^13,66^. *L. chinense* root extracts showed MICs of 0.25–0.5 mg/mL against *S. aureus* and 0.5-1.0 mg/mL against *E. coli*^67^. Our finding of MIC = 0.39 mg/mL for *L. edgeworthii* against *S. aureus* is therefore among the lowest reported for the genus, suggesting that this species may contain particularly potent anti-staphylococcal compounds worthy of isolation and characterization.

The activity against *P. aeruginosa* (MIC = 1.56 mg/mL) is comparable to that reported for *L. europaeum* (1.0–2.0 mg/mL)^43^ and superior to many other plant extracts, which often show no activity against this resistant pathogen at concentrations below 2 mg/mL^68^. This positions *L. edgeworthii* as a valuable resource for anti-pseudomonal drug discovery.

### Implications for multidrug-resistant pathogens and study limitations

The significant activity of *L. edgeworthii* extract against MTCC reference strains, which are known to harbor various resistance mechanisms^24^, suggests potential efficacy against clinical MDR isolates. *S. aureus* MTCC 96 is a standard strain used for susceptibility testing and exhibits methicillin-sensitive characteristics but serves as a suitable model for preliminary screening^69^. *P. aeruginosa* MTCC 424 is known to produce various virulence factors and exhibits inherent resistance to multiple antibiotics^70^. The ability of our extract to inhibit these strains at relatively low concentrations supports the rationale for further testing against clinical MDR isolates.

However, this study has several limitations that must be acknowledged. First, the use of a crude extract means the observed activity is the result of a complex mixture, and the most potent individual compound(s) remain unidentified. Second, the phytochemical analysis was qualitative only; quantitative determination of specific compounds using HPLC, LC-MS, or GC-MS would provide more detailed chemical characterization^71^. Third, the study was conducted only on reference strains; testing against clinical MDR isolates would better reflect real-world efficacy^72^. Fourth, cytotoxicity assays on mammalian cells are essential to evaluate the safety profile of the extract before any therapeutic application can be considered^73^. Fifth, the mechanisms of action proposed are speculative and require experimental validation through specific assays targeting membrane integrity, enzyme inhibition, and nucleic acid synthesis^74^. Sixth, the in vivo efficacy and pharmacokinetics of the extract remain unknown^75^.

### Future perspectives

Based on the promising findings of this preliminary study, several avenues for future research are warranted:

1. *Bioassay-guided fractionation and compound isolation*: The extract should be subjected to chromatographic fractionation (using column chromatography, preparative HPLC) with simultaneous antimicrobial testing to isolate and identify the specific compound(s) responsible for the observed activity, particularly against *S. aureus*^76^. Structural elucidation using NMR, HR-MS, and IR spectroscopy would enable identification of novel bioactive molecules^77^.
2. *Quantitative phytochemical profiling*: Advanced analytical techniques such as HPLC-DAD, LC-MS/MS, and GC-MS should be employed to quantify major bioactive compounds and establish chemical fingerprints for quality control^78^. This would also enable chemotaxonomic comparisons with other *Lycium* species.
3. *Mechanistic studies*: Detailed investigations into the mechanisms of action should include: (i) assessment of membrane integrity using fluorescent probes (e.g., SYTOX Green, propidium iodide)^79^; (ii) measurement of intracellular ATP leakage^80^; (iii) electron microscopy (SEM/TEM) to visualize ultrastructural changes^81^; (iv) evaluation of efflux pump inhibition^82^; and (v) gene expression analysis of key resistance and virulence genes^83^.
4. *Cytotoxicity and safety evaluation*: The extract and isolated compounds should be tested against mammalian cell lines (e.g., human keratinocytes, fibroblasts) using MTT or resazurin-based assays to determine selectivity indices^84^. Hemolytic activity against human erythrocytes should also be assessed^85^. In vivo acute oral toxicity studies in animal models, following OECD guidelines, would establish preliminary safety profiles^86^.
5. *Activity against clinical MDR isolates*: The extract should be tested against a panel of clinical multidrug-resistant isolates, including MRSA, extended-spectrum β-lactamase (ESBL)-producing *E. coli*, carbapenem-resistant *P. aeruginosa*, and other WHO priority pathogens^2^. Synergy studies with conventional antibiotics should be explored to identify potential combination therapies^87^.
6. *Formulation and in vivo efficacy studies*: Development of topical or oral formulations and evaluation of in vivo efficacy using appropriate animal infection models (e.g., murine wound infection, sepsis models) would advance the extract toward clinical application^88^. Pharmacokinetic studies to assess absorption, distribution, metabolism, and excretion are also essential^89^.

## Conclusion

This study provides the first scientific evidence for the antimicrobial potential of *Lycium edgeworthii* leaf extract, thereby addressing a critical knowledge gap for this species within the well-documented *Lycium* genus. The findings conclusively demonstrate that the ethanolic extract is rich in bioactive secondary metabolites including alkaloids, flavonoids, terpenoids, saponins, coumarins, and glycosides aligning with the chemotaxonomic profile of the *Lycium* genus and providing a biochemical basis for its observed activity.

The comprehensive antibacterial evaluation, utilizing complementary qualitative (agar well diffusion) and quantitative (MIC/MBC) methods, revealed that the crude extract possesses significant and broad-spectrum inhibitory effects against both Gram-positive and Gram-negative bacterial pathogens. The most notable result is the extract’s potent activity against *Staphylococcus aureus* (MIC = 0.39 mg/mL), a clinically paramount pathogen responsible for severe community- and hospital-acquired infections, including those caused by methicillin-resistant strains. The concurrent strong activity against *Escherichia coli* (MIC = 0.78 mg/mL) and *Pseudomonas aeruginosa* (MIC = 1.56 mg/mL) further underscores its potential utility against Gram-negative pathogens that are notoriously difficult to treat. The bactericidal nature of the extract (MBC/MIC ratio = 2) adds to its therapeutic relevance.

The observed discrepancy between the large ZOI against *P. aeruginosa* and its higher MIC highlights the importance of employing complementary assays and suggests the presence of compounds with differential diffusion properties, reinforcing the need for bioassay-guided fractionation. The demonstrated activity is likely the result of synergistic interactions among the diverse phytochemicals present, offering a multi-target mechanism that is advantageous in circumventing single-target bacterial resistance.

In summary, this work: (i) provides the first phytochemical characterization of *L. edgeworthii*; (ii) validates its ethnobotanical relevance through scientific evidence of antibacterial activity; (iii) enriches the phytochemical database of the *Lycium* genus; and (iv) conclusively identifies the species as a promising source of antibacterial compounds with efficacy against key drug-resistant pathogens. The logical next steps emerging from this conclusion involve bioassay-guided isolation of the active principle(s), detailed mechanistic studies to elucidate their mode of action, and essential cytotoxicity evaluations to assess therapeutic safety. This research paves the way for the development of *L. edgeworthii*-based antimicrobial agents as part of the strategic response to the global antimicrobial resistance crisis.

## Data Availability

The data that support the findings of this study are available from the corresponding author upon reasonable request.
